# Anemia and the Risk of Cognitive Impairment: An Updated Systematic Review and Meta-Analysis

**DOI:** 10.3390/brainsci11060777

**Published:** 2021-06-11

**Authors:** Woon-Man Kung, Sheng-Po Yuan, Muh-Shi Lin, Chieh-Chen Wu, Md. Mohaimenul Islam, Suleman Atique, Musa Touray, Chu-Ya Huang, Yao-Chin Wang

**Affiliations:** 1 Department of Exercise and Health Promotion, College of Kinesiology and Health, Chinese Culture University, Taipei 11114, Taiwan; nskungwm@yahoo.com.tw (W.-M.K.); drluiswu@gmail.com (C.-C.W.); 2 Graduate Institute of Biomedical Informatics, College of Medical Science and Technology, Taipei Medical University, Taipei 11031, Taiwan; dryuank@gmail.com (S.-P.Y.); 2010rubel@gmail.com (M.M.I.); 3 Department of Otorhinolaryngology, Wan Fang Hospital, Taipei Medical University, Taipei 11600, Taiwan; 4 Department of Otorhinolaryngology, Shuang-Ho Hospital, Taipei Medical University, New Taipei City 23561, Taiwan; 5 Department of Biotechnology and Animal Science, College of Bioresources, National Ilan University, Yilan 26047, Taiwan; neurosurgery2005@yahoo.com.tw; 6 Division of Neurosurgery, Department of Surgery, Kuang Tien General Hospital, Taichung 43303, Taiwan; 7 Department of Biotechnology, College of Medical and Health Care, Hung Kuang University, Taichung 43302, Taiwan; 8 Department of Health Business Administration, College of Medical and Health Care, Hung Kuang University, Taichung 43302, Taiwan; 9 Department of Health Informatics, College of Public Health and Health Informatics, University of Ha’il, Ha’il 55211, Saudi Arabia; gcufpharmd@yahoo.com; 10 Department of Public Health, University of The Gambia, Serrekunda 3530, The Gambia; musatouray@utg.edu.gm; 11 Taiwan College of Healthcare Executives, Taipei 106607, Taiwan; chuya@tche.org.tw; 12 Department of Emergency, Min-Sheng General Hospital, Taoyuan 33044, Taiwan; 13 Graduate Institute of Injury Prevention and Control, College of Public Health, Taipei Medical University, Taipei 11031, Taiwan

**Keywords:** anemia, dementia, Alzheimer’s, cognitive impairment, iron deficiency, meta-analysis

## Abstract

Background: Cognitive impairment is one of the most common, burdensome, and costly disorders in the elderly worldwide. The magnitude of the association between anemia and overall cognitive impairment (OCI) has not been established. Objective: We aimed to update and expand previous evidence of the association between anemia and the risk of OCI. Methods: We conducted an updated systematic review and meta-analysis. We searched electronic databases, including EMBASE, PubMed, and Web of Science for published observational studies and clinical trials between 1 January 1990 and 1 June 2020. We excluded articles that were in the form of a review, letter to editors, short reports, and studies with less than 50 participants. The Preferred Reporting Items for Systematic Reviews and Meta-analyses (PRISMA) guidelines were followed. We estimated summary risk ratios (RRs) with random effects. Results: A total of 20 studies, involving 6558 OCI patients were included. Anemia was significantly associated with an increased risk of OCI (adjusted RR (aRR) 1.39 (95% CI, 1.25–1.55; *p* < 0.001)). In subgroup analysis, anemia was also associated with an increased risk of all-cause dementia (adjusted RR (aRR), 1.39 (95% CI, 1.23–1.56; *p* < 0.001)), Alzheimer’s disease [aRR, 1.59 (95% CI, 1.18–2.13; *p* = 0.002)], and mild cognitive impairment (aRR, 1.36 (95% CI, 1.04–1.78; *p* = 0.02)). Conclusion: This updated meta-analysis shows that patients with anemia appear to have a nearly 1.39-fold risk of developing OCI than those without anemia. The magnitude of this risk underscores the importance of improving anemia patients’ health outcomes, particularly in elderly patients.

## 1. Introduction

The prevalence of cognitive impairment including dementia is increasing worldwide. It is estimated that more than 75 million people will have dementia by 2030 and 131.5 million by 2050 [1,2]. The risk has been increased in people living in low- and middle-income countries, accounting for more than half (58%) of total patients [3]. From a public health perspective, an increased prevalence of dementia can increase the global health burden [4]. Previous studies reported that the global economic burden of dementia is approximately 1 trillion USD including health expenditures, reduced quality of life, morbidity that raised the concern of healthcare policymakers, physicians, and overall society [5].

Numerous epidemiological studies have highlighted an association between anemia and the increased risk of developing overall cognitive impairment (OCI) [6,7]. Although these studies raised public concern and received wide attention from public health policymakers, the interpretation of the association between anemia and OCI provides a lack of credibility [8]. A randomized control trial was not conducted to find their association in which observational studies and systematic reviews have reportedly supported strong associations [9,10]. The biological mechanisms linking anemia to OCI are not fully understood, although several possible hypotheses have been previously proposed. Chronic brain hypoxia related to anemia might contribute to a decline in cognitive function through an increasing accumulation of amyloid-β [11]. Previous evidence shows an increased association between anemia and the progression of white matter disease and cerebral cortical atrophy, which can accelerate cognitive function decline [12]. Moreover, iron deficiency in the brain hampers its neurotransmitter function by interfering with the rate-limiting enzyme [13].

Peters et al. [10] reported an increased risk of dementia in patients with anemia. However, they included limited studies and suggested that further studies needed to be conducted to draw firm conclusions. Furthermore, Kim et al. [9] investigated the association between anemia and cognitive impairment using sixteen observational studies. The authors emphasized that conducting more research was necessary to reach a robust conclusion because the number of prospective cohort studies were not sufficient. Since there are only four prospective cohort studies that have been published on this topic, we conducted an updated systematic review and meta-analysis that addressed the potential association between anemia and dementia risk.

## 2. Materials and Methods

### 2.1. Experimental Approach

The PRISMA (Preferred Reporting Items for Systematic Reviews and Meta-Analyses) guidelines, which are based on the Cochrane Handbook for Systematic Reviews of Interventions, were used to conduct this study (Supplementary Table S1) [14,15].

### 2.2. Search Strategy

We conducted an updated systematic review and meta-analysis. We searched electronic databases including EMBASE, PubMed, and Web of Science for published observational studies and randomized control trials between 1 January 1990 and 1 June 2020, with no language restrictions. We used the search terms “anemia” OR “iron deficiency”, and “dementia”, OR “Alzheimer”, OR “cognitive impairment”. We also searched the bibliography of retrieved articles to obtain relevant articles.

### 2.3. Inclusion Criteria

Studies that assessed the association between anemia and OCI risk and matched all of the following criteria were included: (a) the primary outcome was OCI, (b) were observational or randomized control trials, (c) included at least 50 participants aged over 18 years old, (d) identified OCI patients using the standard diagnostic criteria (Feighner criteria or Research Diagnostic Criteria/DSM-III/DSM-III-R/DSM-IV/DSM-5/ICD-10), and (e) the diagnosis of anemia patients was based on WHO criteria, i.e., men: Hb < 13 g/dL, women: Hb < 12 g/dL, measured in blood samples.

### 2.4. Exclusion Criteria

Studies were excluded if they were (a) published as a case report or case series with a low number of participants (i.e., <50); (b) published as editorial, letters, commentary, review articles, or conference abstracts; (c) provided insufficient information on OCI patient selection, confounding factors, adjusted variables, and effect size; or (d) used the same database with overlapping study durations.

### 2.5. Data Extraction

Required data were retrieved from selected articles by two independent authors (M.M.I. and S.A.). They developed a standard data collection form, checked all of the variables, and collected the needed information to calculate the risk ratio (RR). Afterwards, they retrieved and compiled the following information: (a) study characteristics: the author’s first name, publication year, country, study duration, study design; (b) demographic characteristics: the number of participants, number of male and female patients, and age; (c) the reference standard of dementia diagnosis, and number of dementia patients; and (d) the adjusted effect sizes such as hazard ratios (HRs) and odds ratios (ORs) with 95% confidence intervals (CIs). We considered the adjusted effect sizes because it helps to reduce potential bias.

### 2.6. Study Quality

Study quality was assessed using the Newcastle Ottawa Scale for observational studies. The summary score was used to categorize the study quality into the following two groups: high (NOS score higher than 7) and low (NOS score equal to or lower than 7).

### 2.7. Statistical Analysis

Comprehensive meta-analysis (CMA, V-2) software was used to analyze the data. An adjusted risk ratio (risk ratio was calculated from adjusted effect sizes (aRR)) with the corresponding 95% CI was calculated to show the magnitude of the association between anemia and OCI. However, the random effect model was used to calculate the heterogeneity between the studies. The Cochran Q statistic and inconsistency statistics (I^2^) were considered to examine whether there was significant heterogeneity in their effect sizes. Previous studies were followed to categorize the heterogeneity as low (I^2^ value < 25%), medium (I^2^ value is between 25% and 50%), and high (I^2^ value greater than 50%) [16,17]. Finally, the forest plot was drawn to present the effect size and the funnel plot to introduce publication risk bias.

## 3. Results

### 3.1. Study Selection

**Figure 1:**
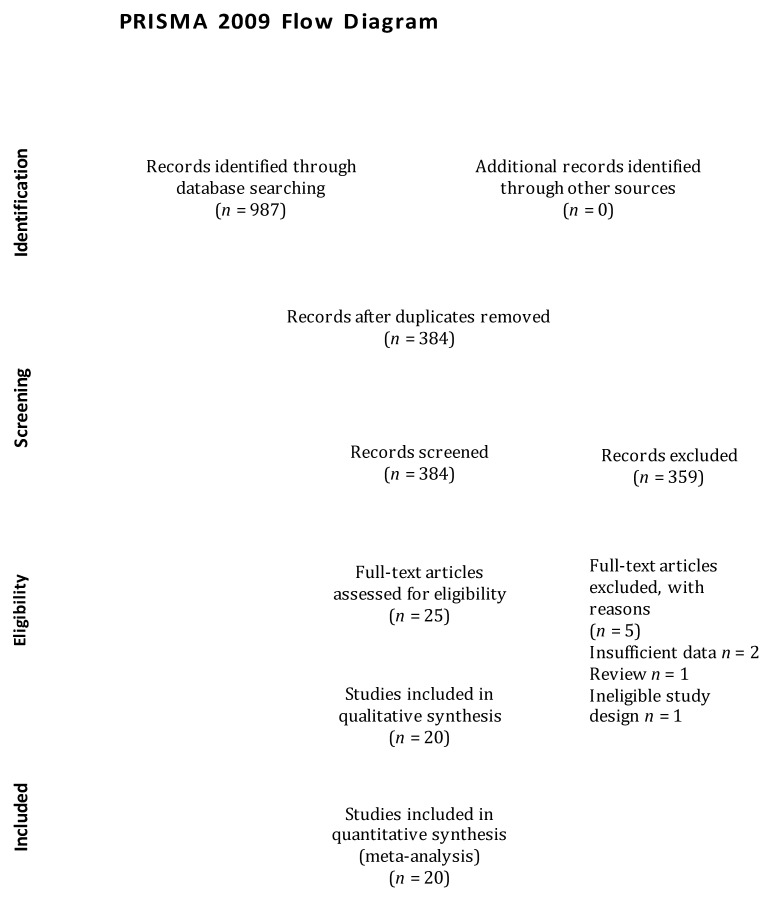
Flow diagram of study selection process.

The articles search was conducted on electronic databases and garnered 987 articles. We reviewed the titles and abstracts of those articles and excluded 962 articles due to a lack of adherence to our pre-specified selection criteria. Afterwards, we screened the full text of the selected 25 articles and assessed the reference lists of relevant articles, retrieving no additional articles. We further excluded 6 articles that did not fulfil the inclusion criteria. Finally, 20 articles were considered in the meta-analysis [6,18,19,20,21,22,23,24,25,26,27,28,29,30,31,32,33,34,35,36], and the flow diagram of the study selection process is illustrated in Figure 1.

### 3.2. Study Characteristics

This updated meta-analysis comprised of 12 cohort studies and 8 case-control studies that are presented in Table 1.

The publication years ranged between 1997 [18] and 2020 [35]. The range of the age of patients was from 35 to 82.5 years. All of the studies selected anemia patients based on WHO criteria, the mean hematocrit, or the lower Hb quintile. Moreover, dementia patients were identified using the Diagnostic and Statistical Manual (of Mental Disorders) (DSM), the Mini-Mental State Examination (MMSE), or the National Institute of Neurological and Communicative Disorders and Stroke, and the Alzheimer’s disease and Related Disorders Association (NINCDS-ADRDA).

### 3.3. Methodological Quality Assessment

We used the Newcastle Ottawa scale (NOS) to evaluate the methodological quality of the included studies, which is recommended by the Cochrane collaboration for nonrandomized studies. The NOS score included case-control and cohort studies ranging from 5 to 8, with a mean score of 7.3.

### 3.4. Meta-Analysis

#### 3.4.1. Anemia and Overall Cognitive Impairment

**Figure 2:**
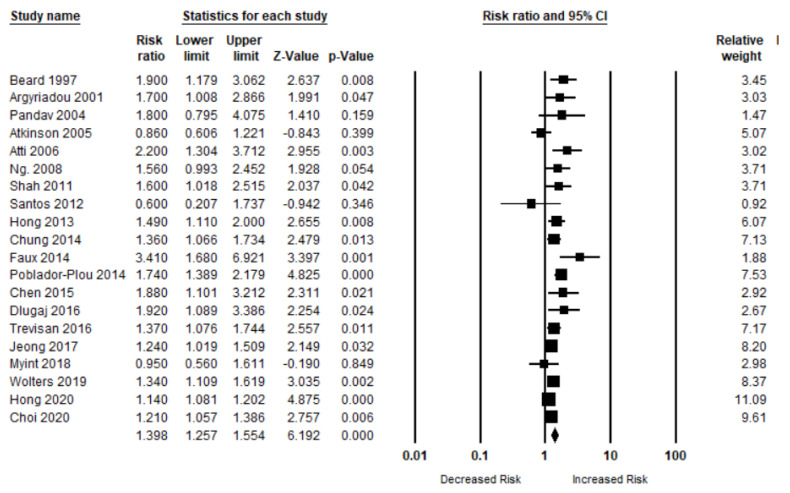
Association between anemia and overall cognitive impairment [6,18,19,20,21,22,23,24,25,26,27,28,29,30,31,32,33,34,35,36].

A total of 20 studies have evaluated the risk of OCI (MCI, AD, and dementia) in patients with anemia. The risk of OCI was significantly higher in patients when compared to those who did not have any kind of anemia (*n* = 20, aRR, 1.39 (95% CI, 1.25–1.55; *p* < 0.001). There was a moderate risk of heterogeneity among the studies (Q = 52.92, I^2^ = 64.10, τ^2^ = 0.02) (Figure 2).

#### 3.4.2. Anemia and All-Cause Dementia Risk

**Figure 3:**
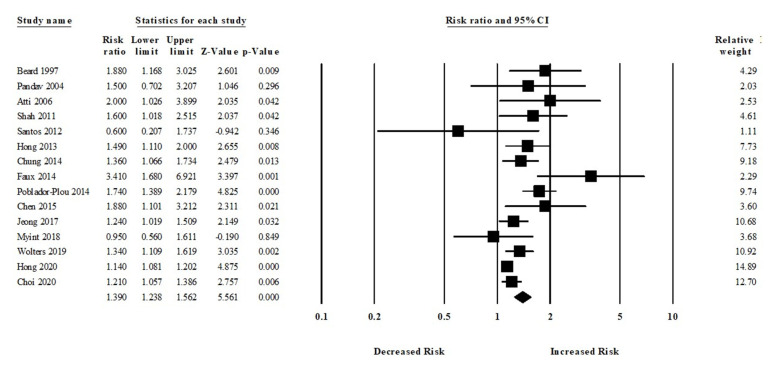
Association between anemia and all-cause dementia [18,19,21,23,24,25,26,27,28,29,32,33,34,35,36].

Among 15 studies, participants who had anemia were more likely to develop all-cause dementia compared to those who did not have any kind of anemia (aRR, 1.39 (95% CI, 1.23–1.56; *p* < 0.001)) (Figure 3). There was a moderate risk of heterogeneity among the studies (Q = 39.47, I^2^ = 64.53, τ^2^ = 0.02).

**Figure 4:**
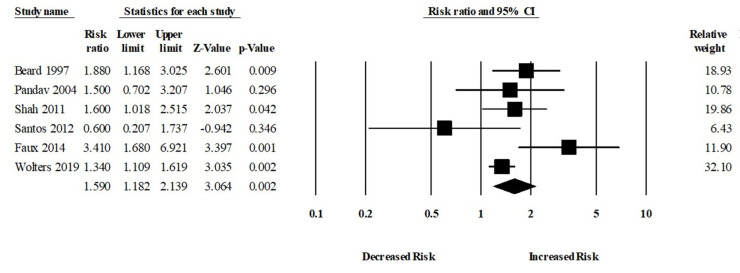
Association between anemia and Alzheimer’s disease [18,19,23,24,27,34].

Moreover, six studies assessed the risk of Alzheimer’s disease (AD) in patients with or without anemia. The patients with anemia were significantly associated with an increased risk of AD (aRR, 1.59 (95% CI, 1.18–2.13; *p* = 0.002)) (Figure 4). There was a moderate risk of heterogeneity among the studies (Q = 10.24, I^2^ = 51.18, τ^2^ = 0.06).

#### 3.4.3. Anemia and MCI Risk

**Figure 5:**
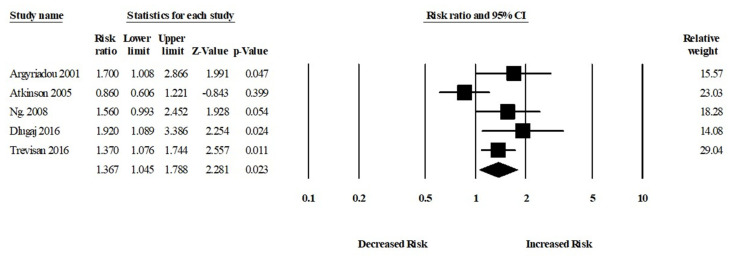
Association between anemia and mild cognitive impairment [6,20,22,30,31].

Among five studies, patients who had anemia were more likely to develop MCI compared to those who did not have any kind of anemia (aRR, 1.36 (95% CI, 1.04–1.78; *p* = 0.02)). There was a moderate risk of heterogeneity among the studies (Q = 8.92, I^2^ = 55.18, τ^2^ = 0.04) (Figure 5).

### 3.5. Subgroup Analysis

We also evaluated the risk of overall cognitive impairment (OCI) in the patients with anemia by methodological quality, study design, and study locations. The subgroup analyses are presented in Table 2.

Eleven high methodological quality studies (based on NOS) examined the risk of OCI in patients with anemia in comparison to patients without anemia. The risk of OCI was higher in patients with anemia (aRR, 1.25 (95% CI, 1.16–1.35; *p* < 0.001)). Nine studies with a low methodological quality assessed the risk of anemia and OCI risk. The overall risk of OCI was significantly higher in patients with anemia (aRR, 1.60 (95% CI, 1.23–2.08; *p* < 0.001)).

In the cohort studies, patients with anemia were more likely to develop OCI when compared to those who did not have anemia ((number of studies: N = 12, aRR, 1.25 (95% CI, 1.14–1.37; *p* < 0.001)). However, the risk of OCI in the patients with anemia was noticeably higher in a case-control study design ((number of studies: *n* = 8, aRR, 1.65 (95% CI, 1.37–2.00; *p* < 0.001)).

Seven European studies demonstrated that patients with anemia were significantly associated with an increased risk of OCI ((number of studies: *n* = 7, aRR, 1.48 (95% CI, 1.28–1.71; *p* < 0.001)). In North America, four studies have evaluated the risk of OCI in patients with anemia. The pooled RR of OCI was 1.37 (95% CI, 0.98–1.92; *p* = 0.06) in patients with anemia. Furthermore, in Asia, nine studies have assessed the risk of OCI in patients with anemia. The pooled RR of OCI was 1.29 (95% CI, 1.13–1.47; *p* < 0.001) in the patients with anemia when compared to those who did not have any kind of anemia.

### 3.6. Sensitivity Analysis

We also calculated the risk of OCI in patients with anemia based on various adjustments such as diabetes, stroke, education, APOE e4, and depression. Table 3 shows a summary of the risk based on the adjusted factors.

### 3.7. Publication Bias

**Figure 6:**
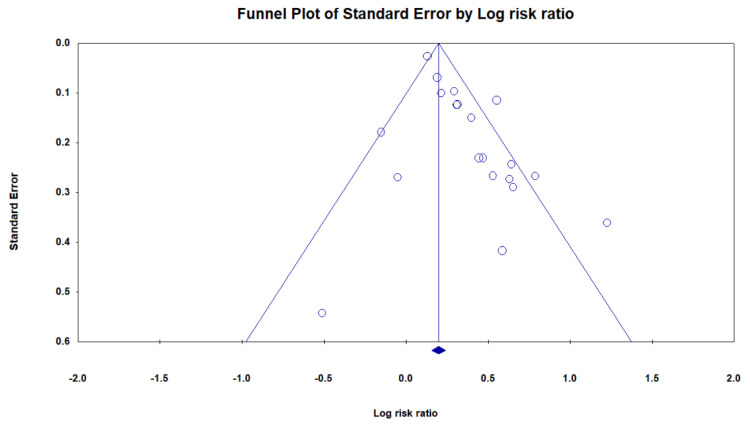
Funnel plot.

We draw the funnel plot to present the clustering of studies below our summary effect estimate (Figure 6). Egger’s test has confirmed the asymmetry of the funnel plot that shows there is no significant publication bias.

## 4. Discussion

### 4.1. Main Findings

The updated meta-analysis of 20 studies with 6558 participants evaluated the association between anemia and dementia. The participants with anemia were significantly associated with an increased risk of OCI including all-cause dementia compared to those who did not have anemia. Moreover, anemia was also associated with an increased risk of Alzheimer’s (1.59-fold). Our findings support previous evidence that anemia increased the risk of OCI [9,10]. The findings of this current meta-analysis were robust because this study shows more subgroup, along with sensitivity, analyses and considered the adjusted effect sizes to summarize the findings. Furthermore, more prospective studies were included, which helps to decrease the potential bias during statistical analyses. A patient with anemia must be followed carefully and routinely monitored for any cognitive abnormalities. Caution should be taken with severely anemic patients where physicians can set a goal for the necessary treatment.

### 4.2. Biological Plausibility

Several biological mechanisms can be used to explain the association between anemia and OCI. First, the most convincing explanation would be related to the limited oxygenation of peripheral tissues due to a low hemoglobin concentration. Previous studies reported that anemia is linked to a decreased cerebral blood flow, leading to OCI [37,38]. A limited cerebral blood flow due to anemia can cause hypoxia; however, prolonged hypoxia changes the iron channels’ excitability and functional expression. The disruption of iron channels then contributes to neurodegeneration [39]. Second, a lower level of oxygen escalates the process of β-amyloid protein formation through the amyloidogenic transformation of the amyloid precursor protein. A higher amount of the β-amyloid protein upregulates native L-type calcium channels and destroys calcium homeostasis [40]. Hyperactivation of calcium expression due to hypoxia in central neurons can lead to the neurotoxicity of the β-amyloid protein and subsequent OCI [41]. Third, a previous study reported an association between anemia and a risk of inflammation [42]; this is one of the main contributors to the underlying physiology of OCI [43]. Increased levels of inflammation are a precursor to cardiovascular disease, which plays an important role in MCI and dementia in later life [44]. A study showed a link between an elevated level of inflammation in middle-aged adults and decreased hippocampal volume, which is correlated with dementia [45]. More studies are warranted to show the exact biological pathway that sees anemia contribute to the development of OCI.

### 4.3. Public Health Implications

Considering the worldwide prevalence of anemia, and the significant association between anemia and OCI risk presented in our updated study, the follow-up of anemia patients with symptoms of MCI could have a considerable effect on public health. Although MCI is mainly asymptomatic, it provides a possibility to prevent the progression of AD or dementia and its complications. Previous studies reported that lifestyle changes could be more effective in preventing OCI, and early psychosocial intervention would be cost effective [46,47]. A randomized controlled trial showed that a complex multimodal activity intervention helped to reduce the risk of OCI in patients who were at a higher risk [48]. Studies also reported that improving a patient’s hemoglobin level and a timely treatment of anemia has a beneficial effect in reducing the risk of progression to OCI [49,50]. Therefore, physicians should encourage patients with iron deficiency anemia to take a replacement therapy with oral or intravenous iron preparations to avoid an unexpected OCI risk.

### 4.4. Strengths and Limitations

Our study has several strengths that need to be addressed. First, it is a comprehensive study that included 20 articles and showed an association between anemia and different types of cognitive impairment risk. Second, the heterogeneity of our study is moderate; therefore, the evidence provided here is less biased. Third, we have considered adjusted effect sizes while collecting our data from the retrieved articles; hence, there is less possibility of confounding factors because they adjusted the possible confounding factors in their studies.

Our study also has several limitations. First, our study could not provide any findings based on the severity of anemia. Second, our study did not provide results based on the duration of anemia and the risk of OCI, although the included studies had at least a one-year follow-up period.

## 5. Conclusions

The main purpose of this updated meta-analysis is to clarify the association between anemia and the risk of OCI. The findings of this study show that anemia is significantly associated with an increased risk of OCI. The early identification of a patient’s conditions and the proper management of anemia patients might contribute to the prevention of OCI risk. Future studies should focus on the biological mechanism of the association between anemia and OCI risk, as well as whether interventions designed to improve hemoglobin levels are effective in reducing OCI risk.

## Figures and Tables

**Table 1:** Study characteristics.

Author Year	Country	Study Design	Female Patients (%)	Mean Age	Criteria for Anemia Patients’ Inclusion	Criteria for Dementia/Cognitive Impairment Patients’ Inclusion	Number of Dementia Patients	OR/HR	NOS
Beard, 1997 [18]	USA	C-C	77.3	82.5	WHO	DSM	191	1.91 (1.19–3.09)	5
Argyriadou, 2001 [6]	Greece	C-C	54.3	65~	Mean hematocrit (Hct): Men < 38%, Women ≤ 36%	MMSE < 24	213	1.70 (1.02–2.90)	6
Pandav, 2004 [19]	India	Co	56.6	69.6	WHO	DSM	26	1.80 (0.8–4.10)	8
Atkinson, 2005 [20]	USA	Co	100	78	WHO	MMSE < 24	68	0.86 (0.60–1.21)	6
Atti, 2006 [21]	Sweden	Co	72	81.5	WHO	DSM	108	2.20 (1.30–3.70)	6
Ng., 2008 [22]	Singapore	C-C	63.2	65.8	Lowest Hb quintile Men: Hb < 13.3 g/dL, Women: Hb < 12.2 g/dL	MMSE < 24	298	1.56 (1.00–2.47)	8
Shah, 2011 [23]	USA	Co	74.7	80.8	WHO	NINCD-ADRDA	113	1.60 (1.02–2.52)	7
Santos, 2012 [24]	Brazil	C-C	60.4	70.4	WHO	DSM	99	0.60 (0.21–1.76)	7
Hong, 2013 [25]	USA	Co	51.8	76.1	WHO	3MS	455	1.49 (1.11–2.00)	8
Chung, 2014 [26]	Taiwan	C-C	54.7	76.4	ICD-9-CM	ICD-9-CM	8300	1.36 (1.07–1.74)	8
Faux, 2014 [27]	Australia	C-C	N/R	35~79	WHO	NINCD-ADRDA	211	3.41 (1.68–6.92)	7
Poblador-Plou, 2014 [28]	Spain	C-C	59.7	75.8	ICD-9-CM	ICD-9-CM	3971	1.74 (1.39–2.18)	7
Chen, 2015 [29]	Taiwan	Co	57.5	38.9	ICD-9-CM	ICD-9-CM	871	1.88 (1.10–3.21)	7
Dlugaj, 2016 [30]	Germany	C-C	50.8	64.4	WHO	IWG	579	1.92 (1.09–3.39)	8
Trevisan, 2016 [31]	Italy	Co	58.4	72.1	Gender-specific Hb tertiles; Men: 13.9–14.9 g/dL, Women: 12.8–13.7 g/dL	MMSE < 24	403	1.37 (1.08–1.75)	8
Jeong, 2017 [32]	S. Korea	Co	51	66	WHO	ICD-9-CM	859	1.24 (1.02–1.51)	8
Myint, 2018 [33]	UK	Co	53	76.5	Hb < 12.9 g/dL	MoCA	655	0.95 (0.56–1.61)	8
Wolters, 2019 [34]	Netherland	Co	64.6	57.7	WHO	MMSE < 26	1520	1.34 (1.11–1.62)	8
Hong, 2020 [35]	Taiwan	Co	60.6	70.4	ICD-9	ICD-9	N/R	1.14 (1.08–1.21)	8
Choi, 2020 [36]	S. Korea	Co	55.8	57.1	WHO	ICD-10	1682	1.21 (1.06–1.39)	8

DSM: Diagnostic and Statistical Manual (of Mental Disorders); Hb: hemoglobin; ICD-9-CM: The International Classification of Diseases, Ninth Revision, Clinical Modification; IWG: International Working Group; MCI: mild cognitive impairment; MMSE: Mini-Mental State Examination; NINCDS-ADRDA: National Institute of Neurological and Communicative Disorders and Stroke and the Alzheimer’s Disease and Related Disorders Association; 3MS: Modified Mini-Mental State; MoCA: the Montreal Cognitive Assessment; NOS: The Newcastle-Ottawa Scale *Diagnosis of anemia was on the basis of WHO criteria, i.e., men: Hb < 13 g/dL, women: Hb < 12 g/dL, measured in blood samples; Co = Cohort; C-C = Case-control; OR = Odds ratio, HR = Hazard ratio, N/A = Not reported.

**Table 2:** Subgroup Analyses.

Subgroup	Pooled Estimate			Test of Heterogeneity			
	N	aRR	*p*-Value	τ^2^	I^2^	Q	*p-*Value
**Outcomes**							
**Overall cognitive impairment (OCI)**							
Study design							
Cohort	12	1.25 (1.14–1.37)	<0.001	0.008	41.63	18.84	0.06
Case-control	8	1.65 (1.37–2.00)	<0.001	0.02	34.91	10.74	0.15
**Study quality**							
High	11	1.25 (1.16–1.35)	<0.001	0.004	29.39	14.16	0.16
Low	9	1.60 (1.23–2.08)	<0.001	0.09	62.71	21.45	0.006
**Location**							
Asia	9	1.29 (1.13–1.47)	<0.001	0.01	56.11	18.22	0.02
Europe	7	1.48 (1.28–1.71)	<0.001	0.009	24.98	7.99	0.23
USA	4	1.37 (0.98–1.92)	0.06	0.07	67.31	9.17	0.02

*n* = Number of studies included in the analysis.

**Table 3:** Sensitivity Analyses.

Adjustment	Pooled Estimate			Test of Heterogeneity			
	N	aRR	*p*-Value	τ^2^	I^2^	Q	*p-*Value
**Overall Cognitive Impairment**							
Diabetes	13	1.37 (1.22–1.54)	<0.001	0.02	61.02	30.79	0.002
Education	11	1.38 (1.20–1.59)	<0.001	0.01	26.04	13.52	0.19
Hypertension	10	1.33 (1.19–1.49)	<0.001	0.01	62.60	24.06	0.004
Smoking	9	1.28 (1.13–1.45)	<0.001	0.01	39.54	13.23	0.10
Depression	6	1.33 (1.17–1.51)	<0.001	0.005	21.71	6.38	0.27
Stroke	5	1.42 (1.18–1.71)	<0.001	0.01	34.52	6.10	0.19
APOE e4	3	1.40 (1.20–1.63)	0.06	0	0	0.58	<0.001
CRP	2	1.38 (1.17–1.63)	<0.001	0	0	0.34	0.55
**Dementia**							
Diabetes	10	1.34 (1.17–1.54)	<0.001	0.02	65.11	25.79	0.002
Education	6	1.41 (1.22–1.63)	<0.001	0	0	4.25	0.51
Hypertension	8	1.32 (1.16–1.49)	<0.001	0.01	67.42	21.49	0.003
Smoking	6	1.28 (1.16–1.41)	<0.001	0.002	11.59	5.65	0.34
Depression	4	1.28 (1.13–1.45)	<0.001	0.003	19.77	3.74	0.29
Stroke	3	1.36 (1.10–1.70)	0.05	0.01	45.42	3.66	0.16
APOE e4	2	1.38 (1.17–1.63)	<0.001	0	0	0.34	0.55
CRP	2	1.38 (1.17–1.63)	<0.001	0	0	0.34	0.55

*n* = Number of studies included in the analysis.

## Data Availability

The data used to support the findings of this study are included within the article.

## Supplementary Materials

The following are available online at https://www.mdpi.com/article/10.3390/brainsci11060777/s1, Supplementary Table S1: PRISMA 2020 Checklist.
